# Location inference for hidden population with online text analysis

**DOI:** 10.1186/s12942-020-00245-x

**Published:** 2020-12-09

**Authors:** Chuchu Liu, Ziqiang Cao, Xin Lu

**Affiliations:** 1 College of Systems Engineering, National University of Defense Technology, Changsha, 410073 China; 2 School of Software Engineering, Shenzhen Institute of Information Technology, Shenzhen, 518172 China

**Keywords:** Location inference, Hidden population, MSM, Text analysis, Geographic distribution

## Abstract

**Background:**

Understanding the geographic distribution of hidden population, such as men who have sex with men (MSM), sex workers, or injecting drug users, are of great importance for the adequate deployment of intervention strategies and public health decision making. However, due to the hard-to-access properties, e.g., lack of a sampling frame, sensitivity issue, reporting error, etc., traditional survey methods are largely limited when studying such populations. With data extracted from the very active online community of MSM in China, in this study we adopt and develop location inferring methods to achieve a high-resolution mapping of users in this community at national level.

**Methods:**

We collect a comprehensive dataset from the largest sub-community related to MSM topics in Baidu Tieba, covering 628,360 MSM-related users. Based on users’ publicly available posts, we evaluate and compare the performances of mainstream location inference algorithms on the online locating problem of Chinese MSM population. To improve the inference accuracy, other approaches in natural language processing are introduced into the location extraction, such as context analysis and pattern recognition. In addition, we develop a hybrid voting algorithm (HVA-LI) by allowing different approaches to vote to determine the best inference results, which guarantees a more effective way on location inference for hidden population.

**Results:**

By comparing the performances of popular inference algorithms, we find that the classic gazetteer-based algorithm has achieved better results. And in the HVA-LI algorithms, the hybrid algorithm consisting of the simple gazetteer-based method and named entity recognition (NER) is proven to be the best to deal with inferring users’ locations disclosed in short texts on online communities, improving the inferring accuracy from 50.3 to 71.3% on the MSM-related dataset.

**Conclusions:**

In this study, we have explored the possibility of location inferring by analyzing textual content posted by online users. A more effective hybrid algorithm, i.e., the Gazetteer & NER algorithm is proposed, which is conducive to overcoming the sparse location labeling problem in user profiles, and can be extended to the inference of geo-statistics for other hidden populations.

## Introduction

A population is “hidden” when no sampling frame exists and public acknowledgment of membership in the population is potentially threatening [1–3]. Examples of hidden populations include men who have sex with men (MSM) [4–6], lesbians [7–9], sex workers (SW) and injecting drug users (IDU). To date, the study of hidden populations has mainly focused on interviews and questionnaire surveys based on nonprobability sampling methods, e.g., snowball sampling [10] and respondent-driven sampling (RDS) [11, 12] While in most cases, these traditional methods are inefficient, limited in sample size and representativeness, and challenged by privacy concerns and reporting errors [13–16]. Besides, with concerns about sensitivity and privacy, hidden populations tend to conceal their personal information, including their locations. There are many difficulties in conducting comprehensive studies on demographic characteristics of hidden populations, especially on their geographic distribution. However, understanding the geolocation distribution of hidden populations and their spatial clustering is crucial to public health management.

According to recent statistics, approximately 58.8% of the world’s population now use the internet [17], and by 2020, there will be around 30 billion devices connected to each other [18]. Nowadays, our daily lives are inseparable from the internet. The numerous data generated on the internet provides opportunities to infer demographic attributes of internet users with computational techniques at an unprecedented scale. Due to social discrimination, hidden populations lack reliable channels for communication and tend not to disclose their information in the real world. Instead, the anonymity of internet provides a good sense of security and has made online social networking prevailing among hidden populations [19, 20], offering alternatives for understanding demographic statistics (gender, age, location, etc.) of traditionally hard-to-access populations.

Location-based services (LBS) are incredibly useful across many domains, including personalized services (e.g. local restaurants, hospitals, events), prompting alert, assessment and emergency responses to disease or disasters, as well as detecting security intrusion, etc. [21]. However, it is still challenging to obtain user location due to the sparse geo-enabled features in social media. Although users from social networks can fill up profiles with their personal information, the use of these data is however limited as it may be subject to large reporting error and many users may opt to make their personal information hidden to the public. It is reported that, on average only 35% of Facebook users declare locations [22], and a large volume of invalid or low precision locations are often submitted. Regarding other ways of geolocation retrieving, user location given by the IP address is not reliable and needs to be continually updated [23]. GPS provides locations with best accuracy and reliability, while many people do not want to disclose their detailed coordinates. For example, in Brazil, under 1% of tweets provide GPS data [24]. Although there has been a large number of studies on location inference with internet data for general population, e.g., through platforms of Twitter [25, 26] Facebook [27, 28], and Flickr [29, 30], there is however very limited studies with such applications on hidden or hard-to-access populations, as large-scale corpus of hidden populations is generally lacking, and the applicability of algorithms is not known when they are to be generalized to a new population.

As a representative of hidden populations, MSM suffers more social pressure and discrimination than the general population in many cultures, and the demographics of them, especially the geographic distribution are of critical importance for public health management and HIV prevention [31, 32]. However, it is still difficult to locate the MSM population. To date, relevant study of this population has mainly relied on sampling estimations based on questionnaire surveys. We thereby take MSM as an example in this study, to test and develop appropriate location inferring approaches based on large corpus of online data. We collected a comprehensive dataset in this study, covering 628,360 MSM-related users from the largest sub-community related to MSM topics, gay-bar, in the world’s largest Chinese community (i.e., Baidu Tieba [33]). With observations in gay-bar, a user’s location is more likely to be mentioned in his/her own posts. Based on users’ publicly available posts, we develop a new hybrid method to infer the geographic locations for online MSM. Compared with other approaches that consider only text content, the hybrid method proposed in this paper can achieve the best performance, improving the inferring accuracy from 50.3 to 71.3%.

## Materials and methods

### Data collection

Baidu Tieba is the largest Chinese online community and it consists of a variety of sub-communities on different topics and gathers massive numbers of user groups with different interests. The gay-bar is the largest sub-community related to MSM in Baidu Tieba, which serves as a community for homosexual friends to seek partners and chat. As of June 14, 2018, gay-bar had 4.67 million followers and more than 300 million posts. The massive user data generated in this open community is of great significance for analyzing the demographic attributes of the MSM population in China. In this study, by using Scrapy [34, 35], a fast web-crawling framework, we elaborately developed a web crawler with Python to extract the data we needed from the webpages. All posts from the latest to the oldest, as well as all following comments and replies under these posts on gay-bar were collected, for a total of 13,023,367 records and 628,360 unique users. All the data was distributed from January 29, 2005, to June 14, 2018.

We also obtained public profile data of active users. A total of 359,438 records was collected, including user name, sex, self-reported location, latitude and longitude coordinate located by GPS, etc. However, among all users, only 8.6% (30,948) declare the location field in their profiles, indicating that over 91% gay-bar users did not fill in their locations when registering. In addition, the authenticity and accuracy of these reported locations needs to be considered as large reporting error may occur due to the privacy concerns of users. In addition, when the user is using a mobile device, he can opt to reveal his GPS coordinates publicly. Among all 359,438 users, we find that about 10.9% had doing so, adding to a complementary dataset for algorithm validation.

### Location inference method with online text analysis

**Fig. 1:**
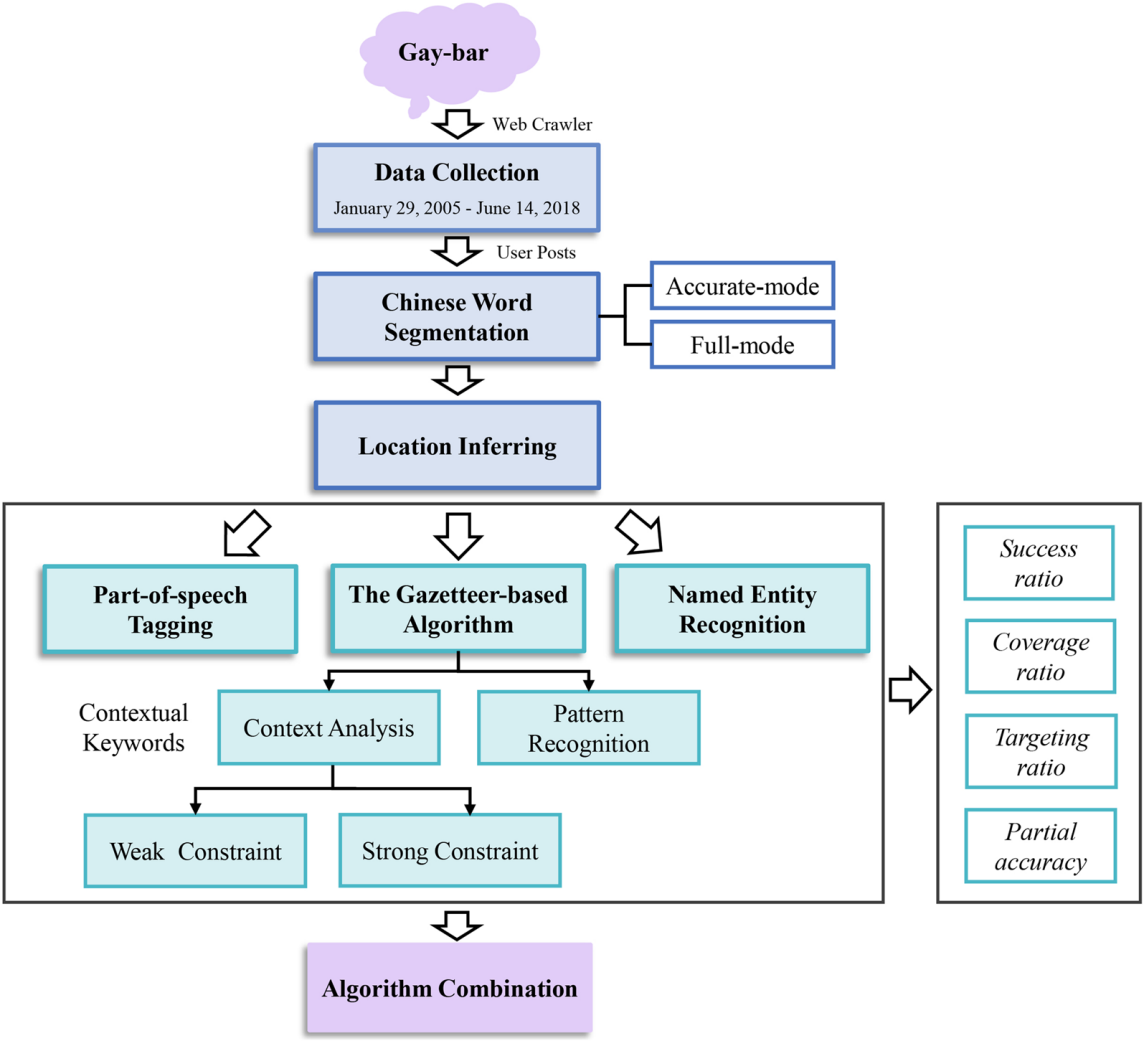
The workflow and links between location inferring algorithms

Observing the text content posted by gay-bar users, we find that the subject of most posts in this community is related to offline dating. In order to find partners or friends with close distance, most users tend to actively reveal their locations when posting online [36], which plays a key role in meeting with each other and finally transferring the relationship from the internet to the real world. Therefore locations revealed in these posts can promise a great authenticity, and provide a new perspective for us to evaluate locations of the MSM population as well as their geographic distribution. Based on the text analysis of gay-bar posts, we evaluate and compare the performances of mainstream location inference algorithms to solve the online locating problem of Chinese MSM population. To improve the inference accuracy, we propose a new hybrid method, which integrates different algorithms by voting on the inference results, and is proven to guarantee a higher accuracy (71.3%) on the location inferring of MSM in online social communities. The workflow and links between algorithms are presented in Fig. 1.

#### Training and test data

In this study, all user posts collected from gay-bar (including posts, comments and replies), as the corpus input of the inferring algorithm, are used to extract specific location information. There are a total of 628,360 unique users in the dataset, of which 156,777 have provided geographic information in their posts at certain degree. However, in many cases these geolocation data are not about where they stay but discussions about POI (point of interests) such as tourist attractions. In order to effectively distinguish whether the geographic information appearing in a user’s posts represents his actual location, we experiment with multiple approaches and compare effects of different algorithms with a testing set. The test data is composed of randomly selected 10,000 users' posts. And each user is manually labelled with his actual geographic location by reading the post content.

#### Location inference algorithms

Current online-corpus location inferring algorithms mainly considers the text associated with each user, including the gazetteer-based method [37], part-of-speech tagging [38, 39] and named entity recognition (NER) [40, 41]. The summary and comparison of the three algorithms are presented in Table 1. Due to the posting characteristics of gay-bar users, we mainly infer locations at the province-level and city-level. In the gazetteer-based location inferring method, we use the popular Chinese word segmentation module, Jieba [42], which is more suitable for Chinese text analysis, to cut the posts into the most accurate segmentations. We also consider unigrams of the post text, and remove all punctuation, stop words and URLs. In this method, the gazetteer words used to match the user location belong to the geographical gazetteer of China [43], where province-level gazetteer words contain provinces, municipalities, autonomous regions, Hong Kong and Macao; city-level gazetteer words contain prefectural-level city, municipalities, Hong Kong and Macao. Besides, in the part-of-speech tagging method, the part-of-speech recognition function of Jieba is employed, where the geographical terms are identified as a particular label (“ns”). And the named entity recognition method is realized by using the Chinese geographic name recognition based on a cascaded hidden Markov model (the HMM-Viterbi algorithm) [44].

**Table 1:** Comparison of mainstream location inferring algorithms

	The Gazetteer-based Method	Part-of-speech (POS) Tagging	Named Entity Recognition (NER)
Features	Identifying geographical names according to external location knowledge (e.g., dictionary containing names of cities and states)	Recognizing geographical terms in a corpus based on the part of speech of its component words, according to both their definitions and contexts	Identifying and classifying words mentioned in unstructured corpus as pre-defined entity classes, i.e., persons, locations, organizations, etc. based on HMM models
Strengths	It is a popular approach when looking for locations in Web text [45]; The algorithm is simple and easy to implement	Part-of-speech information is a pre-requisite in many NLP (Natural Language Processing) algorithms	The algorithm is fast, and suitable for processing large-scale datasets
Limitations	Largely relies on the gazetteer, and easily affected by external geographic databases [46–48]	Vulnerable to linguistic errors and idiosyncratic style [38]; Algorithm accuracy is relatively low	Cannot identify names of local streets or buildings, non-standard place abbreviations and misspellings which are common in microtext

#### Improvement on existing algorithms

When a user mentions a geographic name in the post, we find that it does not necessarily refer to the location where the user is or has been or will go, that is, his track. To improve the accuracy of the inference algorithm, we try to combine other approaches in natural language processing with the location extraction, such as context analysis and pattern recognition.

##### Context keyword analysis

The context keyword analysis is added to determine whether the geographic information in the post refers to a user’s track. In this paper, eight Chinese keywords, i.e., ‘坐标’ ('coordinate' in English), ‘定位’ ('location'), ‘在’ ('in'), ‘是’ ('am'), ‘同’ ('same'), ‘求’ ('seek'), ‘人’ ('from'), and ‘交友’ ('dating') are selected to filter the geolocation words. The appearance of a context keyword in the post is then considered as an improved likelihood of referring actual location for the user.

##### Pattern recognition on post sentence

In order to further restrict the syntax patterns in user corpus and strengthen the filtering rules, the idea of pattern recognition is introduced. According to the expression characteristics of gay-bar users, 7 typical modes are defined, i.e., keywords before location (former keywords), keywords after location (later keywords), global keywords, individual location, individual location with punctuations or symbols, location with modal particle and province with city. These modes can cover most of syntax that MSM use when referring to their locations. Keywords employed in pattern recognition are shown in Table 2.

**Table 2:** Chinese keywords employed in pattern recognition

	Former keywords	Later keywords	Global keywords
In Chinese	坐标,定位,同,在,从,去,是,也是,求,就是,大…	人,上学,上班,有,有吗,附近,的,滴,是,加…	交友,大学,学院,公司,同城,私聊…
In English	Coordinate, location, same, in, from, come, am, also be, seek, big (usually describe someone’s own place), etc	Person, go to school, work, have, any, close, is, add, follow, etc	Making friends, university, college, company, same city, private chat, etc

#### Algorithm measurements

Since a user's location may consist of several geographic places, such as his hometown, the province or city where he works or studies. Meanwhile the same user is likely to migrate to different places at different time. A set is used to record locations of each user. Therefore we cannot simply measure the algorithm results with the precision and recall used in binary classification problems. In order to comprehensively judge the performances of different approaches, four new indexes are defined in this paper, namely absolute accuracy (success rate, *S* for short), coverage ratio (*C*), targeting ratio (*T*), and partial accuracy (*P*).

The absolute accuracy measures whether the algorithm result is exactly the same as the manually labeled result, $S = N_{s} /N_{all}$, where $N_{all}$ is the number of all test users, and $N_{s}$ is the number of users whose locations are accurately inferred.

The coverage ratio measures the comprehensiveness of an algorithm. When the results from manual annotation are completely included in the inference results, the algorithm are believed to achieve a complete coverage. It is defined as $C = N_{c} /N_{all}$, where $N_{c}$ is the number of users whose locations are totally excavated by the algorithm.

The targeting ratio is used to determine whether all geographic words recognized by the algorithm are correct, $T = N_{T} /N_{all}$, where $N_{T}$ is the number of users whose inferred results from the algorithm are all correct even if some manual labels may not be covered.

The partial accuracy is used to judge whether inference locations have any intersections with manual labels, $P = N_{P} /N_{all}$, where $N_{P}$ is the number of users whose locations are partly inferred.

By comparing the inference set evaluated by the algorithm with the manual label set, these four measurements are employed to determine the algorithm with the best performance. *S* is the most important indicator used to measure the algorithm effect.

## Results

### Performances of different text-based location inferring algorithms

In this study, all online posts from 156,777 users who mentioned geographic information are used as the corpus input in location inferring algorithms. As the first step, three mainstream algorithms are applied to determine the most suitable approach regarding the location inference for online hidden population. The performances of three different approaches, i.e., the gazetteer-based method, part-of-speech tagging and Chinese NER, are compared. Accuracy of the inference results from different algorithms is shown in Table 3.

**Table 3:** The performances of three location inferring algorithms

	Gazetteer	Part-of-speech	Chinese NER
*S*	**0.503**	0.352	0.487
*C*	**0.932**	0.892	0.927
*T*	**0.518**	0.370	0.502
*P*	**0.966**	0.945	0.964

As we can see, the gazetteer-based method achieves the highest accuracy on all measurements, suggesting that it is more suitable for extracting the location information from short texts, e.g., user posts on gay-bar. Other algorithms, such as part-of-speech tagging and NER, which are more widely used in location inferring from Chinese texts nowadays, are not so effective than the traditional gazetteer-based method instead. The latter proves to be the simplest, fastest, and most effective.

However, the success rate (*S*) obtained by the gazetteer-based algorithm is only 0.503, which means that only 50.3% users’ locations are fully identified without any errors. In order to further improve the performance of this algorithm, context analysis and pattern recognition is introduced to the gazetteer-based method. From Table 4, we can see that the addition of contextual keyword analysis can improve the success rate and targeting ratio of the algorithm, whereas the stricter rules of pattern recognition do not achieve a good performance. This is because that the latter method defines more specific grammar, syntax, keywords as well as keyword positions to filter text, with more restrictions on user posting. Due to the variety of Chinese expressions, especially in internet language, the syntax patterns used by online MSM are so flexible, leading to a lower accuracy of the algorithm with pattern recognition.

**Table 4:** The performance of the gazetteer-based method after strengthen filtering rules

	Gazetteer	Gazetteer with context analysis	Gazetteer with pattern recognition
*S*	0.503	**0.512**	0.493
*C*	0.932	**0.929**	0.733
*T*	0.518	**0.528**	0.667
*P*	0.966	**0.965**	0.800

Overall, for the social network whose users mainly focus on making friends or dating, such as gay-bar, there are numerous obvious geographic information exposed in the short text of user posts. And the gazetteer-based method combined with the contextual keyword analysis is more effective in user location inference, by which over 51.2% users’ tracks can be absolutely correctly recognized.

### Improvement of the gazetteer-based algorithm

The comparison above illustrates that the traditional gazetteer-based location inferring method with context analysis can achieve better performance in the gay-bar dataset. However, the highest accuracy is still at a relatively low level, therefore, we aim to further improve the algorithm by considering more conditions.

#### Different constraints on contextual keywords

In this section, we try to change the way which keywords are constrained to improve the algorithm performance. Two constraints (weak or strong) are mainly considered. The weak constraint is that as long as any keyword appears in any post of a user, all geographic words in his posts are considered to be his possible locations. For analysis above, the weak constraint on the context keywords is used by default. We attempt to replace the weak constraint with the strong constraint to explore whether the algorithm accuracy would be improved as a result. In detail, the geographic word must appear with any context keyword in a same post, then this geographical term can be considered to be possible user location. The performance of the algorithm with different constraints is shown in Table 5.

**Table 5:** The performance of the gazetteer-based method with strong constraint

	Gazetteer	Gazetteer with context analysis (weak)	Gazetteer with context analysis (strong)
*S*	0.503	0.512	**0.542**
*C*	0.932	0.929	**0.762**
*T*	0.518	0.528	**0.614**
*P*	0.966	0.965	**0.866**

It can be seen that compared with weak constraint, the success rate (0.542) and targeting ratio (0.614) of the algorithm both increase after introducing the strong constraint, while the coverage ratio would decrease, i.e., the comprehensiveness of the algorithm results has reduced. The strong constraint of keywords is helpful to improve the correctness of location inference, which is more suitable for situations aiming at locating accuracy. Although the accuracy of weak constraint is relatively low, the algorithm can achieve more comprehensive results ($C = 0.929$). And user actual locations are mostly covered by the results of inference algorithm, which is more conducive to identifying all geographic tracks of MSM.

#### Expanding contextual keyword set

As the context analysis applied in the location inferring algorithm above only utilizes eight keywords, i.e., *‘coordinate’, ‘location’, ‘in’, ‘am’, ‘same’, ‘seek’, ‘from’ and ‘date’*. By further analyzing the posting characteristics of gay-bar users, other ten common keywords are added, including *‘school’, ‘work’, ‘friends’*, etc., for the purpose of covering more posts referring to user locations. The algorithm performance after expanding contextual keywords is shown in Table 6.

**Table 6:** The algorithm performance with keyword augmentation

	Strong constraint	Strong constraint with more keywords	Strong constraint (full-mode segmentation)
*S*	0.542	**0.540**	**0.513**
*C*	0.762	**0.780**	**0.756**
*T*	0.614	**0.604**	**0.582**
*P*	0.866	**0.878**	**0.854**

It shows that adding keywords has no significant effect on improving the accuracy of location inferring. Instead, since more contextual keywords tend to broaden the permission on syntax, constraints on geographic information are insufficient, which reduces the performance of the algorithm.

#### Different word segmentation mode

In this study, the word segmentation method used in the gazetteer-based algorithm is from Jieba, a popular Chinese word segmentation package. All previous results are based on the accurate-mode of Jieba, which aims at cutting the Chinese sentence most accurately. We attempt to change the word segmentation method and examine whether the accuracy of the algorithm can be improved. In this section, the full-mode word segmentation method [49] is used, which scans all possible words that can be formed in a sentence. With observing the change of measurements, we compare the effect of the two different word segmentation methods on the location inference.

As shown in Table 6, the results prove that although the full-mode word segmentation can increase the number of words from the text, it may mislead the location inferring and reduce the accuracy of the algorithm as the result of the word ambiguity. Compared with the full-mode method, the accurate-mode word segmentation is more helpful for the gazetteer-based location inference.

### Hybrid voting algorithm on location inference (HVA-LI)

To further improve the accuracy of location inference for hidden population, while maintaining the superiority of existing algorithms, we adopt the ensemble learning approach and develop a hybrid voting algorithm, called HVA-LI, by allowing different approaches to vote to determine the best inference results. The main goal of this hybrid method is by setting multiple filters to improve the inference accuracy.

#### Gazetteer-based algorithm and part-of-speech tagging (Gazetteer & PT)

In addition to the gazetteer-based algorithm with context analysis (strong constraint), which achieves the highest success rate, different inference algorithms are considered to be introduced to work together. In this section, the most superior algorithm (S-Gazetteer, for short) tend to be combined with the part-of-speech tagging algorithm (PT, for short). Both algorithms calculate each user's corpus to extract possible locations and determine the final output by voting together. Locations that both algorithms agree on are considered the final inference results.

#### Gazetteer-based algorithm and NER (Gazetteer & NER)

In order to further verify the superiority of the ensemble learning approach, the Gazetteer algorithm is designed to effectively combine with the NER algorithm. Similarly, all final outputs are determined by two algorithms’ voting on their results. However, the difference is that in this section two different combinations are carried out. Firstly, without the introduction of context analysis, a simple ensemble of the basic gazetteer-based method (Gazetteer) and NER is employed as the basic benchmark. We then add the context analysis with strong constraint, which is proven to be the best strategy to improve the algorithm accuracy in the gazetteer-based methods, into the simple ensemble algorithm to examine the changes in algorithm performance.

#### Performances of the algorithm combinations

**Fig. 2:**
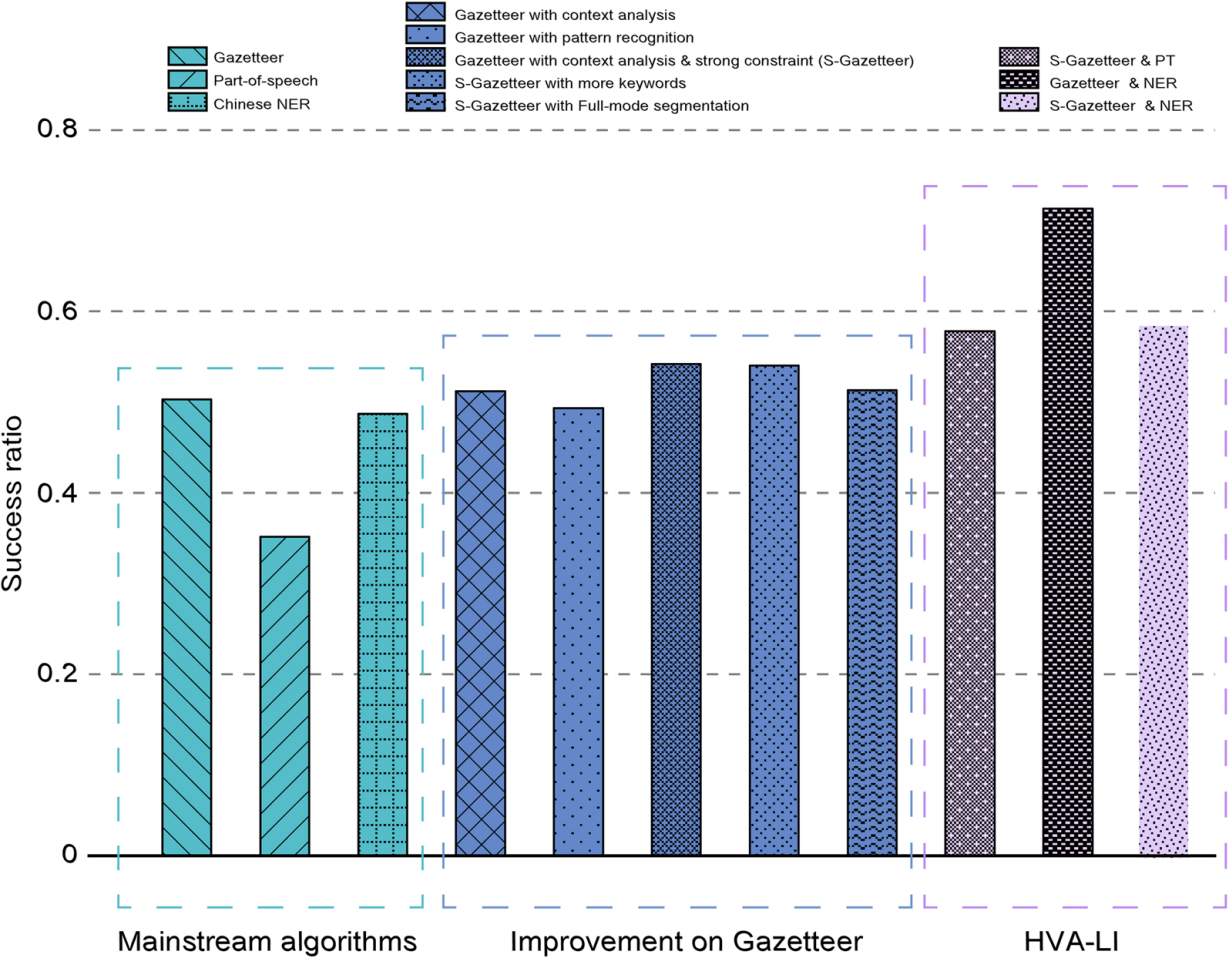
Absolute accuracy of location inferring algorithms

The performances of different algorithm combinations is shown in Table 7. It can be seen that the success rates of all inference algorithms have improved, compared with the best algorithm before combination (S-Gazetteer$,{ }S = 0.542$). The hybrid voting algorithm (HVA-LI) can be considered more effective in location inferring for online MSM. Significantly, it can be seen that the ensemble of the basic gazetteer-based method and NER can achieve a much higher accuracy, and the success rate for location inference is up to 0.713, approximately 32% higher than the best algorithm without combination. Surprisingly, however, the introduction of context analysis does not improve the effectiveness of the algorithm. We find that the success rate ($S = 0.586$) decreases dramatically in comparison to the former simple combination. This may be because the contextual keywords destroy the integrity of post texts, hence degrades the performance of NER as well as the accuracy of the algorithm. The absolute accuracy of all algorithms evaluated in this study is shown in Fig. 2.

**Table 7:** The performance of HVA-LI

	S-Gazetteer & PT	Gazetteer & NER	S-Gazetteer & NER
*S*	0.578	**0.713**	0.586
*C*	0.756	**0.713**	0.767
*T*	0.666	**0.914**	0.668
*P*	0.883	**0.914**	0.881

### Distribution of online MSM in China

#### MSM distribution extracted from user profiles

**Fig. 3:**
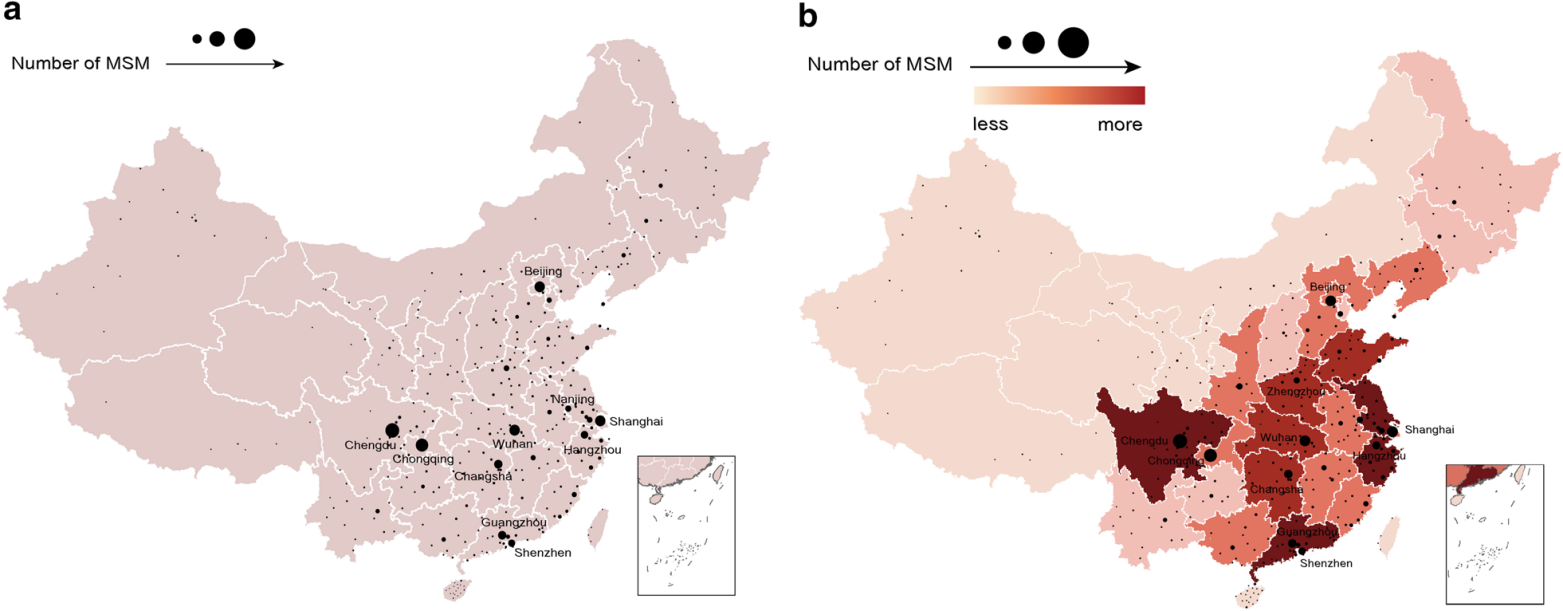
**a** The city distribution of gay-bar users from location fields in profiles, **b** the location distribution extracted from the GPS coordinates of gay-bar users on both the city-level and the province-level

The public profile data provides us with a direct way to obtain users’ locations. We collected all possible profiles of active users in gay-bar, for a total of 359,438 records, in which about 10% of user location data (including GPS coordinates) is not empty. Figure 3A shows the city distribution of user geo-locations reported in profiles of gay-bar users. It can be seen that most of the users are from cities of Chengdu, Chongqing, Wuhan, Shanghai, Beijing, Changsha, and Guangzhou. And we find that the number of users is not entirely related to the population of the city.

The city distribution and province distribution resolved from the latest recorded latitude and longitude coordinates of gay-bar users are shown in Fig. 3b. It can be seen that the top 5 provinces are Guangdong, Sichuan, Jiangsu, Zhejiang and Hunan. The corresponding top 5 cities are Chengdu, Chongqing, Shanghai, Wuhan and Beijing with most gay-bar users, which is basically the same as the location distribution filled in user profiles above. Through further analysis, it is found that those users who were willing to provide their GPS coordinates were more likely to fill in the location fields correctly in the registration profiles.

#### MSM distribution inferred by the Gazetteer & NER algorithm

**Fig. 4:**
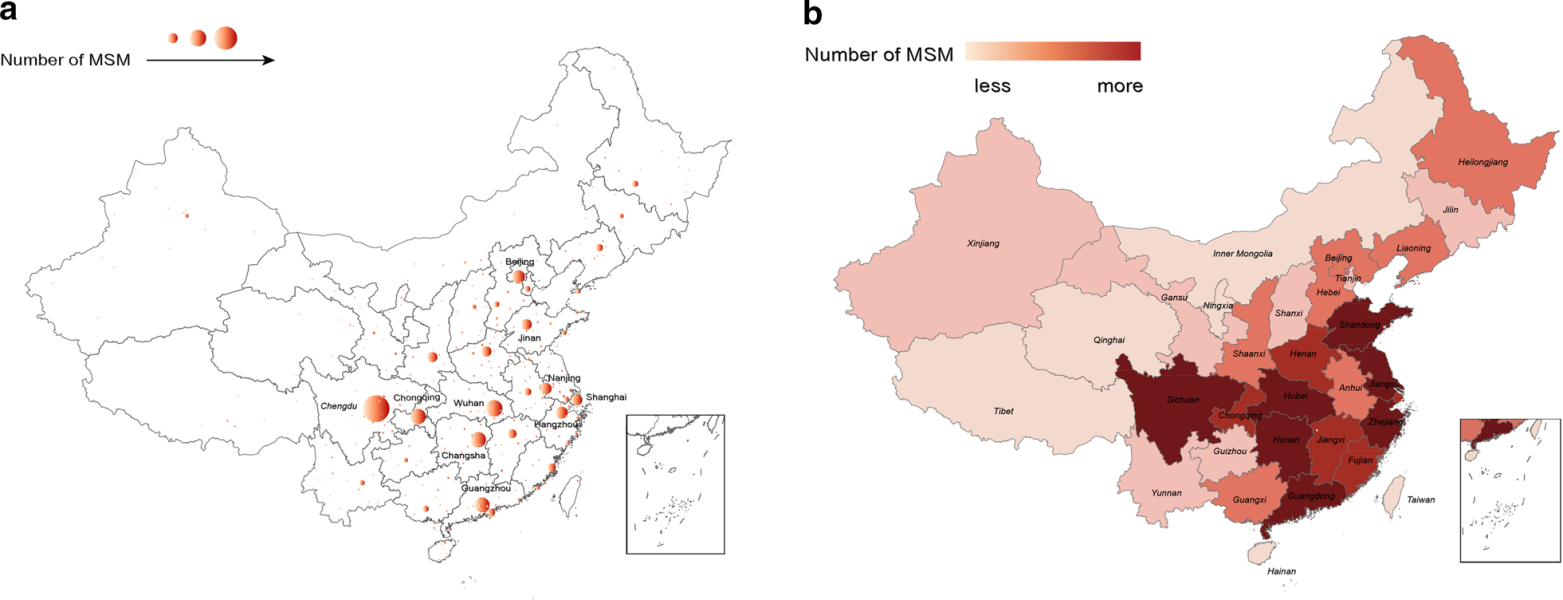
The geographic distribution of gay-bar users inferred from the published posts. **a** City-level distribution. **b** Province-level distribution

According to recent locations disclosed in text content posted by gay-bar users, we use the Gazetteer & NER algorithm to estimate the geographic distribution of online MSM in China, which covers over 156,000 MSM-related users. It makes up for the missing data, as well as the incomplete and inaccurate location information on user profiles, providing a good solution to the hard-to-access properties of hidden populations. As shown in Fig. 4, it can be seen that most MSM-related users are from cities of Chengdu, Wuhan, Chongqing, Changsha, Guangzhou and Beijing. And the top 5 provinces with most relevant users are Sichuan, Guangdong, Zhejiang, Jiangsu and Hunan. These results are consistent with statistics of Chinese MSM population in recent studies [50, 51], indicating that MSM are mainly distributed in large cities in the eastern and southwestern China which is more economically developed and culturally open. Compared with the location distribution extracted from user profiles, MSM distribution inferred by the algorithm is more accurate. In addition to covering more users, our algorithm can infer users’ more recent whereabouts revealed in their posts, while location fields filled in profiles when users register can be out of date and less reliable.

## Conclusion & Discussion

For social networking platforms orient the online-to-offline dating, users tend to expose their geographic information, as well as other basic demographic characteristics in the text content of their posts, offering an unprecedented opportunity for the statistical inference on demographic attributes of relevant populations. In this study, we try to compare mainstream location inference algorithms and develop more efficient approaches to infer the geolocation distribution of the hidden population. Among the popular location inferring methods, the classic gazetteer-based algorithm has achieved better results. Meanwhile this algorithm has other advantages, such as fast calculation speed and easy implementation. We have proposed a few amendments to the gazetteer-based algorithm by introducing the context analysis as well as the strong constraint on contextual keywords. In addition, we develop a hybrid voting algorithm (HVA-LI) by allowing different approaches to vote to determine the best inference results, which guarantees a more effective way on location inference for hidden population. Significantly, the hybrid algorithm consisting of the simple gazetteer-based method and named entity recognition (NER) is proven to be the best choice to deal with inferring users’ locations disclosed in short texts on online communities, which achieves the best accuracy ($S = 0.713$) on the MSM-related dataset, much higher than those from existing popular algorithms, whose best accuracy is 0.503.

In summary, in order to expand the availability of the geolocation information of users in social networks, especially for online hidden population, we have explored the possibility of location inferring by analyzing textual content posted by online users. And we have proposed a more effective hybrid algorithm, i.e., the Gazetteer & NER algorithm, to largely increase the accuracy of location inference for hidden population and to overcome the sparseness problem of dealing with user profile data. These more adequate and accurate geo-location information can be widely adopted in the fields of disease control and population mobility analysis, which are important to the public health management. Significantly, benefiting from locating high-risk populations (e.g., MSM, sex workers), decision-makers and health service providers can better understand the spatial distribution of relevant groups, so as to develop more efficient strategies for mitigating infectious diseases (e.g., HIV and STDs). In addition, associating the geographic region in user posts with their temporal series can provide important clues regarding travel, migration and displacement.

The methodology used in this work can also be extended to the location inferring of other social networks. And our proposed algorithm has other applications beyond the specific MSM case study presented here. But currently, the calculation process of the method is largely dependent on the text content of users’ posts. When the geographical words mentioned are sparse, the inference accuracy tends to be reduced. Therefore, the algorithm is much more suitable for the location inference on certain populations whose purpose of posting is finding partners or seeking friends. In future work, we plan to implement more sophisticated inferring models, by incorporating other sources of information, e.g., the friendship network of online users, to further improve the performance and application of the location inference algorithm.

## Data Availability

All data analyzed in this study are publicly available, all posts in the datasets can be collected on the website of Baidu Tieba (https://tieba.baidu.com/f?kw=gay). Other data that support the findings in this study are available from the corresponding author on reasonable request.

## References

[1] Liu C, Lu X (2019). Network evolution of a large online msm dating community: 2005–2018. Int J Env Res Pub He.

[2] Liu C, Lu X (2018). Analyzing hidden populations online: topic, emotion, and social network of HIV-related users in the largest Chinese online community. BMC Medical Inform Decis Mak.

[3] Berghe WV, Nöstlinger C, Hospers H (2013). Laga M International mobility, sexual behaviour and HIV-related characteristics of men who have sex with men residing in Belgium. BMC Public Health.

[4] Jie W, Ciyong L, Xueqing D, Hui W, Lingyao H (2012). A syndemic of psychosocial problems places the MSM (men who have sex with men) population at greater risk of HIV infection. PLoS ONE.

[5] Berghe WV, Nöstlinger C, Hospers H, Laga M (2013). International mobility, sexual behaviour and HIV-related characteristics of men who have sex with men residing in Belgium. BMC Public Health.

[6] Huang G, Cai M, Lu X (2019). Inferring opinions and behavioral characteristics of gay men with large scale multilingual text from blued. Int J Env Res Pub He.

[7] Ren Z, Howe CQ, Zhang W (2019). Maintaining, “mianzi” and “lizi”: Understanding the reasons for formality marriages between gay men and lesbians in China. Transcult Psychiatry.

[8] Wen G, Zheng L (2020). Relationship status and marital intention among chinese gay men and lesbians: the influences of minority stress and culture-specific stress. Arch Sex Behav.

[9] Flage A (2019). Discrimination against gays and lesbians in hiring decisions: a meta-analysis. Int J Manpow.

[10] Baltar F, Brunet I (2012). Social research 2.0: virtual snowball sampling method using Facebook. Internet Res.

[11] Chen S, Lu X (2017). An immunization strategy for hidden populations. Sci Rep.

[12] Lu X. Respondent-driven sampling: theory, limitations & improvements. 1st edn. Stockholm: Karolinska Institutet; 2013.

[13] Lu X (2013). Linked ego networks: improving estimate reliability and validity with respondent-driven sampling. Soc Netw.

[14] Jia Z, Mao Y, Zhang F, Ruan Y, Ma Y, Li J (2013). Antiretroviral therapy to prevent HIV transmission in serodiscordant couples in China (2003–11): a national observational cohort study. Lancet.

[15] Magnani R, Sabin K, Saidel T, Heckathorn D (2005). Review of sampling hard-to-reach and hidden populations for HIV surveillance. Aids.

[16] Lu X, Malmros J, Liljeros F, Britton T (2013). Respondent-driven sampling on directed networks. Electron J Stat.

[17] World Internet Users Statistics and 2020 World population stats. 2020. https://www.internetworldstats.com/stats.htm. Accessed 3 Mar 2020.

[18] Nordrum A. Popular internet of things forecast of 50 billion devices by 2020 is Outdated. https://spectrum.ieee.org/tech-talk/telecom/internet/popular-internet-of-things-forecast-of-50-billion-devices-by-2020-is-outdated. Accessed 18 Aug 2016.

[19] Bien CH, Best JM, Muessig KE, Wei C, Han L, Tucker JD (2015). Gay apps for seeking sex partners in China: implications for MSM sexual health. AIDS Behav.

[20] Young LE, Michaels S, Jonas A, Khanna AS, Skaathun B, Morgan E (2017). Sex behaviors as social cues motivating social venue patronage among young black men who have sex with men. AIDS Behav.

[21] Hinds J, Joinson AN (2018). What demographic attributes do our digital footprints reveal?. A systematic review. PloS one..

[22] Gundecha P, Barbier G, Liu H. Exploiting vulnerability to secure user privacy on a social networking site. In: Proceedings of the 17th ACM SIGKDD international conference on Knowledge discovery and data mining; 2011. 10.1145/2020408.2020489

[23] Rodrigues E, Assunção R, Pappa GL, Renno D, Meira W (2016). Exploring multiple evidence to infer users’ location in Twitter. Neurocomputing.

[24] Davis CA, Pappa GL, de Oliveira DRR, de Arcanjo FL (2011). Inferring the location of twitter messages based on user relationships. Trans GIS..

[25] Ajao O, Hong J, Liu W (2015). A survey of location inference techniques on Twitter. J Inf Sci.

[26] Pontes T, Magno G, Vasconcelos M, Gupta A, Almeida J, Kumaraguru P, et al. Beware of what you share: Inferring home location in social networks. In: 2012 IEEE 12th International conference on data mining workshops; 2012. 10.1109/ICDMW.2012.106.

[27] Chaabane A, Acs G, Kaafar MA. You are what you like! information leakage through users’ interests. In: Proceedings of the 19th annual network & distributed system security symposium (NDSS); 2012. https://researchers.mq.edu.au/en/publications/you-are-what-you-like-information-leakage-through-users-interests. Accessed 1 Feb 2012.

[28] Backstrom L, Sun E, Marlow C. Find me if you can: improving geographical prediction with social and spatial proximity. In: Proceedings of the 19th international conference on World wide web; 2010. 10.1145/1772690.1772698.

[29] Popescu A, Grefenstette G. Mining User Home Location and Gender from Flickr Tags. Washington: ICWSM; 2010. p. 307–310.

[30] Zheng D, Hu T, You Q, Kautz H, Luo J. Towards lifestyle understanding: Predicting home and vacation locations from user's online photo collections. In: Ninth international AAAI conference on web and social media; 2015. Citeseer.

[31] Beyrer C, Baral SD, Van Griensven F, Goodreau SM, Chariyalertsak S, Wirtz AL (2012). Global epidemiology of HIV infection in men who have sex with men. Lancet.

[32] Qi J, Zhang D, Fu X, Li C, Meng S, Dai M (2015). High risks of HIV transmission for men who have sex with men—a comparison of risk factors of HIV infection among MSM associated with recruitment channels in 15 cities of China. PLoS ONE.

[33] Wikipedia. The introduction of Baidu Tieba.2020. https://en.wikipedia.org/wiki/Baidu_Tieba. Accessed 4 November 2020.

[34] Han X, Zheng L. Design and implementation of firmware data acquisition system based on scrapy framework. In: 2020 IEEE international conference on power, intelligent computing and systems (ICPICS). 2020. 10.1109/ICPICS50287.2020.9202251.

[35] Kaiying D, Senpeng C, Jingwei D (2020). On optimisation of web crawler system on Scrapy framework. Int J Wirel Mob Comput.

[36] Bruch EE, Newman M (2019). Structure of online dating markets in US cities. Sociol Sci.

[37] Wang X, Xu M, Ren Y, Xu J, Zhang H, Zheng N (2014). A location inferring model based on tweets and bilateral follow friends. JCP.

[38] Derczynski L, Ritter A, Clark S, Bontcheva K. Twitter part-of-speech tagging for all: Overcoming sparse and noisy data. In: Proceedings of the international conference recent advances in natural language processing RANLP 2013. https://www.aclweb.org/anthology/R13-1026/. Accessed 9 Sep 2013.

[39] Owoputi O, O’Connor B, Dyer C, Gimpel K, Schneider N, Smith NA. Improved part-of-speech tagging for online conversational text with word clusters. In: Proceedings of the 2013 conference of the North American chapter of the association for computational linguistics: human language technologies. https://www.aclweb.org/anthology/N13-1039/. Accessed 9 Jun 2013.

[40] Lozano MG, Schreiber J, Brynielsson J (2017). Tracking geographical locations using a geo-aware topic model for analyzing social media data. Decis Support Syst.

[41] Gelernter J, Mushegian N (2011). Geo-parsing messages from microtext. Trans. GIS.

[42] Pypi. Project description.2020. https://pypi.org/project/jieba/. Accessed 20 Jan 2020.

[43] The geographical gazetteer of China. https://www.china.com.cn/ch-quhua/.

[44] Yu H-K, Zhang H-P, Liu Q, Lv X-Q, Shi S-C (2006). Chinese named entity identification using cascaded hidden Markov model. J China Inst Commun..

[45] Amitay E, Har'El N, Sivan R, Soffer A. Web-a-where: geotagging web content. In: Proceedings of the 27th annual international ACM SIGIR conference on research and development in information retrieval. 2004. 10.1145/1008992.1009040.

[46] Wang K, Yu W, Yang S, Wu M, Hu Y, Li S (2015). Location inference method in online social media with big data. Ruan Jian Xue Bao J Softw.

[47] Hecht B, Hong L, Suh B, Chi EH, eds. Tweets from Justin Bieber's heart: the dynamics of the location field in user profiles. In: Proceedings of the SIGCHI conference on human factors in computing systems; 2011. 10.1145/1978942.1978976.

[48] Eisenstein J, O’Connor B, Smith NA, Xing E, eds. A latent variable model for geographic lexical variation. In: Proceedings of the 2010 conference on empirical methods in natural language processing; 2010. https://www.aclweb.org/anthology/D10-1124. Accessed Oct 2020.

[49] Github. Module description on Chinese word segmentation of Jieba.2020. https://github.com/fxsjy/jieba. Accessed 15 Feb 2020.

[50] Hu M, Xu C, Wang J (2020). Spatiotemporal analysis of men who have sex with men in mainland China: soc app capture-recapture method. JMIR mHealth uHealth.

[51] Huang D, Wang J, Yang T (2020). Mapping the spatial-temporal distribution and migration patterns of men who have sex with men in mainland China: a web-based study. Int J Environ Res Public Health.

